# Trends and patterns of modern contraceptive use and relationships with high-risk births and child mortality in Burkina Faso

**DOI:** 10.3402/gha.v8.29736

**Published:** 2015-11-09

**Authors:** Abdoulaye Maïga, Sennen Hounton, Agbessi Amouzou, Akanni Akinyemi, Solomon Shiferaw, Banza Baya, Dalomi Bahan, Aluisio J. D. Barros, Neff Walker, Howard Friedman

**Affiliations:** 1 Centre for Demographic Research, Université Catholique de Louvain, Louvain-la-Neuve, Belgium; 2 Institut Supérieur des Sciences de la Population, Ouagadougou University, Ouagadougou, Burkina Faso; 3 United Nations Population Fund, New York, NY, USA; 4 United Nations Children's Fund, New York, NY, USA; 5 Demographic and Social Statistics Department, Obafemi Awolowo University, Ife, Nigeria; 6 School of Public Health, Addis Ababa University, Addis Ababa, Ethiopia; 7 Institut National de la Statistique et de la Démographie, Ouagadougou, Burkina Faso; 8 United Nations Population Fund, Ouagadougou, Burkina Faso; 9 International Center for Equity in Health, Federal University of Pelotas, Pelotas, Brazil; 10 Institute for International Programs, Bloomberg School of Public Health, Johns Hopkins University, Baltimore, MD, USA

**Keywords:** modern contraceptive use, high-risk births, fertility, under-five child mortality, Burkina Faso

## Abstract

**Background:**

In sub-Saharan Africa, few studies have stressed the importance of spatial heterogeneity analysis in modern contraceptive use and the relationships with high-risk births.

**Objective:**

This paper aims to analyse the association between modern contraceptive use, distribution of birth risk, and under-five child mortality at both national and regional levels in Burkina Faso.

**Design:**

The last three Demographic and Health Surveys – conducted in Burkina Faso in 1998, 2003, and 2010 – enabled descriptions of differentials, trends, and associations between modern contraceptive use, total fertility rates (TFR), and factors associated with high-risk births and under-five child mortality. Multivariate models, adjusted by covariates of cultural and socio-economic background and contact with health system, were used to investigate the relationship between birth risk factors and modern contraceptive prevalence rates (mCPR).

**Results:**

Overall, Burkina Faso's modern contraception level remains low (15.4% in 2010), despite significant increases during the last decade. However, there are substantial variations in mCPR by region, and health facility contact was positively associated with mCPR increase. Women's fertility history and cultural and socio-economic background were also significant factors in predicting use of modern contraception. Low modern contraceptive use is associated with higher birth risks and increased child mortality. This association is stronger in the Sahel, Est, and Sud-Ouest regions. Even though all factors in high-risk births were associated with under-five mortality, it should be stressed that short birth spacing ranked as the highest risk in relation to mortality of children.

**Conclusions:**

Programmes that target sub-national differentials and leverage women's health system contacts to inform women about family planning opportunities may be effective in improving coverage, quality, and equity of modern contraceptive use. Improving the demand satisfied for modern contraception may result in a reduction in the percentage of women experiencing high-risk births and may also reduce child mortality.

**Paper context**

Modern contraceptive use is one of the most studied issues in reproductive health, but few things are known about differentials at the sub-national level and their association with risk-high birth and child mortality. Despite a low modern contraceptive use rate in Burkina Faso, regional disparities can reflect an issue of equity in terms of family planning (FP) coverage. However, women's contact with the health system may represent a critical opportunity for promoting and increasing contraceptive use. Furthermore, improving the modern contraceptive use can result in greater birth spacing and may contribute to a reduction in the child mortality rate.

Despite some notable advances in the past few decades in improving access to and involvement in FP, the percentage of demand for modern contraception satisfied remains low in most sub-Saharan countries, along with high rates of maternal, neonatal, and child mortality. The average total fertility rate (TFR) in Burkina Faso declined slightly from 6.8 children per woman (1998) to 6.0 (2010), but remains one of the highest in the world. Although the maternal mortality ratio has nearly halved since 1990, from 770 maternal deaths per 100,000 live births (1990) to 400 (2013), the ratio is still high (1).

Even though child mortality rates have dropped significantly in recent decades, the country is still suffering the burden of high childhood mortality. The under-five mortality rate in Burkina Faso was 129 per 1,000 births between 2005 and 2010 (2), while the most common causes of mortality in 2013 included malaria, neonatal causes, pneumonia, and diarrhoea (3). Mortality rates have been linked to mothers’ fertility characteristics, such as birth spacing, parity, or mothers’ age at childbirth (4, 5).

The sub-Saharan African region is characterised by a comparatively strong demand for larger families (6), and it has experienced less fertility decline than other regions of the world (7, 8). One of the key proximate determinants of fertility (9) is low contraceptive use, especially the use of modern contraceptive methods (6). Overall, about 15% of women in a union used modern contraceptive methods and 23.8% had an unmet need for FP in 2010 in Burkina Faso (2). This low contraception use is characteristic of many countries in sub-Saharan Africa, where there are estimated to be approximately 14 million unplanned pregnancies annually (10). However, there is substantial variation in contraceptive use both between and within countries. For instance, contraceptive prevalence was, on average, 15% in western Africa, while the rate exceeded 33% in some eastern African countries (11). At the sub-national level, contraceptive prevalence rates are generally lower in rural areas as compared to urban areas, generally resulting in higher fertility rates being observed in rural areas (12).

In addition, low contraceptive use may result in increased mortality risks for both mothers and children. Indeed, an increased contraceptive use would have reduced by 40 percentage points the risk of maternal death within the last 20 years and improved child survival by reducing the risks of prematurity or low weight at birth as a result of lengthening the interval between births in developing countries (13). The association between contraceptive use and mortality is indirectly observed through fertility factors such as early births (mother's age at birth <18 years), late births (mother's age at birth >34 years), short birth spacing (<24 months between births), and higher birth order or parity (>3). Births in which any (or a combination) of these indicators are present are referred to as *high-risk births* (14, 15), that is, births that represent a potential increased mortality risk to both mothers and children.

The low contraceptive use and subsequent difficulties in fertility regulation are explained in the literature by various barriers. Contraceptive method–related reasons have been identified and may relate to geographic and financial impediments to contraceptive access or FP services, including a limited choice of methods or experiencing side effects (16–18). There are medical barriers, such as the quality of services, providers’ biases, or medical and legal restrictions regarding access to certain methods (17–19). In some contexts, there are network barriers through social myths and misconceptions around contraception and partner and family approval of contraceptive decisions (20–22). Similarly, women's status in traditional society, place of residence, religious influence, social taboos, and cultural beliefs about high fertility represent potential sociocultural factors affecting modern contraceptive use (18, 22, 23). At the individual level, there are barriers related to women's background and around the quality of information about FP, the fear of adverse effects or the shame associated with affordability of particular methods, contact with health facilities, and women's sociodemographic characteristics (age, education, employment, marital status, parity, etc.) (16, 17, 21, 22).

Sociodemographics are the most studied of all factors explaining modern contraceptive use or non-use, as required variables are available in most data sources such as the Demographic and Health Surveys (DHS). Other barriers are often analysed through studies using mainly quantitative data from other specific cross-sectional surveys (20), and from qualitative data collection (23, 21).

Even though many investigations of modern contraceptive issues have been undertaken, few studies have shown an interest in exploring the relationship of birth risk and spatial heterogeneity. In this regard, the objective of this paper is to investigate the association between modern contraceptive use, distribution of birth risk, and under-five child mortality both nationally and regionally in Burkina Faso. Specifically, we describe differences in modern contraceptive use, TFR, and high-risk births over the time period of 1998–2010. The paper also investigates the association between modern contraceptive use, categories of high-risk births, and under-five child mortality. Lastly, the determinants or factors likely to be predictive of modern contraceptive use are explored at the national and sub-national levels.

## Methods

### Data and variables

The analyses were performed using data from the last three DHS, conducted in Burkina Faso in 1998, 2003, and 2010, which were representative at both the national and regional levels, available as secondary data. There were five administrative regions in the data set from the survey carried out in 1998. Following a national administrative re-districting in July of 2001, the 2003 and 2010 surveys each contained information for 13 regions. For consistency, we conducted comparison over time by administrative regions using only the last two surveys (2003 and 2010). However, comparisons over time were performed using all three surveys by stratifying by place of residence (urban vs. rural). In this respect, it is worth stressing that Burkina Faso is a predominantly rural country, with only 23% of the population living in urban areas. Except for the Centre (15%) and Hauts-Bassins (62%) regions, which contain the two largest cities (Ouagadougou and Bobo-Dioulasso), the percentage of rural population in the other regions ranges between 81 and 93%; the Sahel and Est regions are the most rural (24). In Burkina Faso, about 60% of citizens are Muslims, in rural areas as well as urban areas. Muslims are more prevalent in the Nord (80%) and Sahel (96%) regions while the biggest proportions of Christians are in the Centre-Ouest (38%) and Centre (42%) regions (24). There are about 60 ethnic groups in Burkina Faso, but almost half of the population belongs to the Mossi group, who primarily live in the Plateau Central, Centre, Centre-Nord, Nord, and Centre-Ouest regions. The Peulh/Fulfulde (mainly in the Sahel and Nord regions), Bobo (in the Hauts-Bassins region), and Gourmantche (in the Est region) are the other important ethnic groups (2).

The primary outcome of interest was the use of any modern contraceptive method at the time of the survey (current use). According to the definition commonly used in the DHS, modern contraceptive methods include the following: female sterilisation, male sterilisation, use of the contraceptive pill, intrauterine contraceptive devices, injectables, implants, female condoms, male condoms, diaphragms, contraceptive foam, contraceptive jelly, lactational amenorrhoea method, or other country-specific modern methods (25). The population base for assessing the coverage of these methods was women in a union (married or living with a partner at the time of the survey), aged 15–49 years old.

Concerning children, many studies have stressed the impact of birth spacing and, indirectly, of modern contraceptive use on perinatal outcomes, neonatal mortality, and deaths among children in the first year of life (26, 27). However, both infancy (<1 year) and early childhood periods (1–4 years) have been the major focus of other studies concerning birth interval and child survival relationships (4, 28). According to an analysis by Cleland et al. (13), the risk of death would fall by 10 and 21 percentage points in infancy and early childhood, respectively, if all childbirths were spaced by at least 2 years. In view of this evidence, we used the entire under-five group for analysis in this paper.

To examine the distribution of high-risk births, we explored the subset of women who had had at least one child within the 5 years preceding the survey. Births were assigned according to their risk factors (mother's age at the time of each childbirth, birth order, and birth spacing in relation to the previous birth) into the 11 categories commonly used (2, 14). These different categories are summarised in Table 1. Births can also be grouped into broader categories (i.e. single high risk or multiple high risk); combining single high risk and multiple high risk gives the category ‘any high risk’. For analyses where we considered the last childbirth for each woman in the preceding 5 years, birth order naturally corresponded to parity of births as well.

**Table 1:** Categories of risks related to fertility behaviour

Category	Definition	Abbreviation
No risk		
Not in any risk category	Second and third birth order born to mother between age 18 and 34 years and birth interval >23 months	No risk
Unavoidable risk		
First birth at age 18–34 years	First child born to mother between ages 18 and 34 years	1st birth+MA=18–34 y
Single high risk		
Mother's age <18 years	Mother's age at birth <18 years, birth order <4, and birth interval >23 months	MA<18 y+BO<4+BI>23 m
Mother's age >34 years	Mother's age at birth >34 years, birth order <4, and birth interval >23 months	MA>34 y+BO<4+BI>23 m
Birth interval <24 months	Birth interval <24 months, mother's age at birth 18–34 years, and birth order <4	BI<24 m+MA=18–34 y+BO<4
Birth order >3	Birth order >3, mother's age at birth 18–34 years, and birth interval >23 months	BO>3+MA=18–34 y+BI>23 m
Multiple high risk		
Mother's age <18 years and birth interval <24 months	Mother's age at birth <18 years and birth interval <24 months or mother's age at birth <18 years and birth order >3	MA<18 y+(BI<24 m∣BO>3)
Mother's age >34 years and birth interval <24 months	Mother's age at birth >34 years, birth interval <24 months and birth order <4	MA>34 y+BI<24 m+BO<4
Mother's age >34 years and birth order >3	Mother's age at birth >34 years, birth order >3, and birth interval >23 months	MA>34 y+BO>3+BI>23 m
Birth interval <24 months and birth order >3	Birth interval <24 months, birth order >3, and mother's age at birth 18–34 years	BI<24 m+BO>3+MA=18–34 y
Mother's age at birth >34 years, birth interval <24 months, and birth order >3	Mother's age at birth >34 years, birth interval <24 months, and birth order >3	MA>34 y+BI<24 m+BO>3

MA, mother's age at childbirth (y, years); BI, birth interval (m, months); BO, birth order.

In addition, covariates at the individual, household, and ecological levels were considered as potential confounders in regression analyses (see Supplementary file). These variables included households’ wealth quintile, mothers’ education level, mothers’ ethnicity, mothers’ religion, and mothers’ age at the time of the survey. Ecological covariates were place of residence (urban vs. rural) and the 13 administrative regions. We also used, as a covariate, the time since last childbirth, corresponding to the age of the youngest child at the time of the survey.

To look for a possible influence of gender preference on contraceptive use, we had covariates related to the number of surviving male and female children born to each woman in the 5 years preceding the survey. Contact with the health system regarding contraceptive use was reflected by indicators such as 1) whether a woman undertook a postnatal health facility visit within 2 months after the last birth and 2) whether a woman had received information about FP during a health facility visit within the last 12 months.

### Analysis

We analysed data using Stata 13.1 software (29). According to the survey design, all the analyses were run on weighted data where the Taylor linearisation series (TLS) (30, 31) was used for variance estimation. Descriptive statistics were used to describe patterns and trends of contraceptive use, distribution of high-risk births and under-five child mortality, and the interrelationships between these variables. To explore the relationship between contraceptive use, distribution of high-risk births, and under-five mortality at the regional level, we performed a time-series analysis where we first calculated women's modern contraceptive prevalence rates (mCPR) by region in 2003. Using 2003 as the reference year, fertility was observed for the next 5 years (until 2008). The regional distribution of high-risk births and the distribution of under-five mortality rates were computed for this same time period (see Figs. 4–6). A variance-weighted regression of TFRs was used to estimate the average annual rate of change (AAR) in fertility.

These analyses were carried out using the three surveys and considered both the national and sub-national levels. From the latest DHS (2010), we used multiple variable logistic regression models, adjusting for other covariates, to explore how high-risk birth recently experienced by women may have influenced modern contraceptive use afterwards. The high-risk birth factors were related to the characteristics of the latest childbirth for each woman between 2005 and 2010. Three regression models were developed, corresponding to rural areas, urban areas, and the entire population, for which adjusted odds ratios (aOR), 95% confidence intervals (95% CI), and *p*-values were reported.

## Results

### Descriptive analyses

#### Patterns and trends of modern contraceptive use

**Fig. 1:**
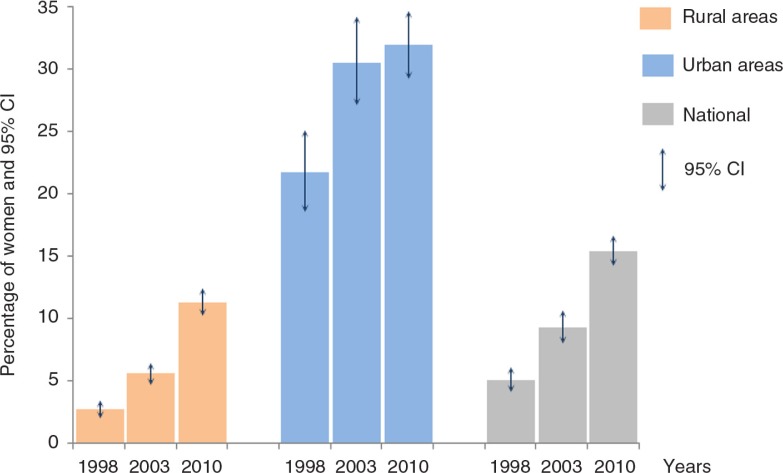
Modern contraceptive use by women in a union by year and by location of residence in Burkina Faso.

In Burkina Faso, the mCPR by women in a union with at least one child tripled from 1998 to 2010 (5.0–15.4%), yet still remained low (see Fig. 1). Women in rural areas had lower mCPR than those in urban areas across all time points. The mCPR in rural areas rose from 2.7% in 1998 to 11.3% in 2010. The largest absolute increase occurred in urban areas between 1998 and 2003, where there was an increase of nearly 10 percentage points. Indeed, 3 out of 10 urban women used a modern method in 2003, compared with 2 out of 10 in 1998. Nevertheless, we did not observe a significant change in modern contraceptive use in urban areas between 2003 and 2010, although the latest survey indicated that urban mCPR remained higher compared with the prevalence in rural areas.

At the regional level (looking at administrative regions), we observed great disparities in modern contraception use between regions (see Table 2). In the latest survey (2010), mCPR was less than 10% in 4 out of 13 regions: Sahel (7.5%), Centre-Est (9.3%), Centre-Nord, and Centre-Ouest (9.8%). Generally speaking, the regions with the lowest mCPRs were more likely to be rural, poorer, have higher illiteracy rates, and belong to the Muslim or animist faiths. The highest mCPRs were observed in the Centre (31.3%) and Hauts-Bassins (28.1%) regions, which host Burkina Faso's political and economic capitals. From 2003 to 2010, contraception prevalence changed significantly in only four regions: Hauts-Bassins, Centre-Sud, Plateau Central, and Est. In Hauts-Bassins, mCPR increased by 16.0 percentage points during that time, while Centre-Sud, Plateau Central, and Est recorded gains of 11.5, 9.8, and 8.9 percentage points, respectively. Nationally, modern contraceptive use increased by 6.1 percentage points.

**Table 2:** Distribution of women in a union with at least one child by regional modern contraceptive prevalence rates and change over time

	mCPR 2003		mCPR 2010		mCPR change 2003–2010		Unweighted number	
				
Region	%	95% CI	%	95% CI	%	Sig.	2003	2010
Boucle du Mouhoun	11.0	(7.5–16.0)	12.1	(9.0–16.1)	1.1	ns	697	1,429
Cascades	13.8	(9.9–19.0)	18.8	(15.3–22.9)	5.0	ns	293	495
Centre	27.1	(21.6–33.4)	31.3	(27.5–35.4)	4.2	ns	791	1,337
Centre-Est	5.5	(3.4–8.9)	9.3	(7.0–12.2)	3.8	ns	747	964
Centre-Nord	7.3	(5.1–10.3)	9.8	(7.7–12.5)	2.5	ns	814	965
Centre-Ouest	6.5	(4.7–9.0)	9.8	(6.9–13.9)	3.3	ns	631	987
Centre-Sud	5.6	(4.2–7.5)	17.1	(13.8–20.9)	11.5	**	517	587
Est	2.5	(1.3–4.9)	11.4	(9.3–14.1)	8.9	**	724	1,199
Hauts-Bassins	12.1	(8.3–17.2)	28.1	(24.3–32.3)	16.0	**	1,111	1,409
Nord	8.9	(6.0–13.1)	10.8	(8.2–14.0)	1.9	ns	924	935
Plateau Central	5.1	(3.2–8.0)	14.9	(11.8–18.6)	9.8	**	453	610
Sahel	3.5	(1.6–7.2)	7.5	(5.1–10.8)	4.0	ns	656	1,014
Sud-Ouest	7.5	(4.0–13.7)	10.6	(8.0–13.9)	3.1	ns	431	537
Total	9.3	(8.0–10.7)	15.4	(14.2–16.7)	6.1	**	8,788	12,467

** Change significant at *p*<0.05; ns, change not significant at *p*<0.05; mCPR, modern contraceptive prevalence rates; CI, confidence interval; ns, not significant; sig., significance.

#### TFR and distribution of births by high-risk factors

Burkina Faso, with a TFR of 6.0 children per woman in 2010 (see Table 3), is among the countries with the highest fertility rates in the world. From 1998 to 2010, the AAR showed a significant but very small decrease in TFRs, barely −0.06 children per woman a year. The annual rate of fertility decline was greater over the 1998–2003 period (−0.11 children per woman a year), compared to the 2003–2010 period, where the AAR was not significant. Contrary to the change over time, there was substantial variation in the TFR according to place of residence and administrative region. In the 2010 survey, the TFR in rural areas (6.8 children per woman) was nearly double that of urban areas (3.9 children per woman). On the basis of administrative region (see Fig. 3), the TFR ranged from a low of 3.7 children per woman (Centre) to a high of 7.5 children per woman in the Sahel and Est regions. As expected, there was a negative relationship between contraception use and TFRs, where regions with the highest mCPRs had the lowest TFRs and vice versa.

**Table 3:** Trends of total fertility rates and standard errors of total fertility rates by location of residence in Burkina Faso

	Rural areas		Urban areas		Nationwide	
			
Year	TFR	SE-TFR	TFR	SE-TFR	TFR	SE-TFR
1998	7.31	(0.10)	4.05	(0.16)	6.80	(0.11)
2003	6.86	(0.10)	3.74	(0.20)	6.24	(0.13)
2010	6.85	(0.07)	3.92	(0.13)	6.09	(0.10)

TFR, total fertility rates; SE-TFR, standard errors of TFR.

The distribution of births by high-risk categories showed that 38% of births occurring between 2005 and 2010 were characterised by a single high-risk fertility behaviour, while 20.3% of births had multiple high-risk factors. In other words, nearly 6 out of 10 births (58.3%) had at least one high-risk factor (see Table 4). High parity birth (birth order >3) was the most substantial high risk factor in the single risk category, consistent with the observation that Burkina Faso has a high TFR. Short birth spacing (<24 months) was an important factor too, but it appeared more commonly as part of multiple high-risk factors. Within the multiple high-risk category, the most common combination was late birth (mother's age >34) combined with short birth spacing (13.8% of births), followed by the combination of short birth spacing with high parity birth (4.8%). The distribution of factors for high-risk birth exhibited no significant changes in the rate of single high-risk factors over time (2010 vs. 1998). Within multiple high-risk categories, only the category combining late birth with high parity birth recorded a slight significant decrease in rate (−2.0 percentage points) during this period.

**Table 4:** Distribution of births by risk category, changes over time, and relationships to under-five child mortality

	Births in the 5 years preceding each survey (%)			Under-five mortality rates in the 5 years preceding each survey (per 1,000)		Unweighted number of births	
			
Birth risk categories	2010	Change	1998–2010	2010	Change 1998–2010	1998	2010
Not in any high risk category	28.2	4.7	**	101.7	−94.7	1,410	4,177
Unavoidable risk category							
First birth between ages 18 and 34 years	13.4	1.2	ns	106.0	−143.3	730	1,985
Single high risk category							
Mother's age <18 years	4.6	−0.9	ns	161.7	−123.5	330	677
Mother's age >34 years	0.5	−0.2	ns	91.8	91.8	15	79
Birth interval <24 months	3.8	−0.9	ns	190.6	−43.1	280	569
Birth order >3	29.0	0.1	ns	123.6	−57.5	1,737	4,296
*Subtotal*	*38.0*	−*1.3*	ns	*134.9*	−*68.7*	2,363	5,620
Multiple high risk category							
Age <18 years and birth interval <24 months	[0.2]	[−0.5]	**	[291.6]	[75.3]	41	31
Age >34 years and birth interval <24 months	#	#	#	#	#	2	5
Age >34 years and birth order >3	13.8	−1.7	ns	112.1	−85.1	932	2,045
Birth interval <24 months and birth order >3	4.8	−2.0	**	212.4	−78.6	410	714
Age >34 years and birth interval <24 months and birth order >3	1.4	−0.6	ns	270.1	−5.4	119	212
*Subtotal*	*20.3*	−*4.7*	**	*155.1*	−*79.9*	1,504	3,006
In any high-risk category	58.3	−6.1	**	142.0	−73.9	3,867	8,626
Total	100	–		126.2	−89.4	6,008	14,789

** Change significant at *p*<0.05; ns, change not significant at *p*<0.05; [*x*], estimates based on unweighted cases between 25 and 50; #, estimates based on fewer than 25 unweighted cases.

**Fig. 2:**
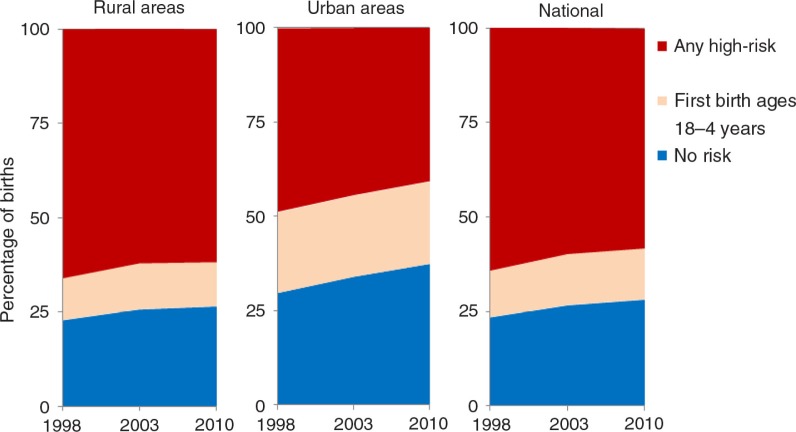
Distribution of high-risk births by location of residence over time in Burkina Faso.

**Fig. 3:**
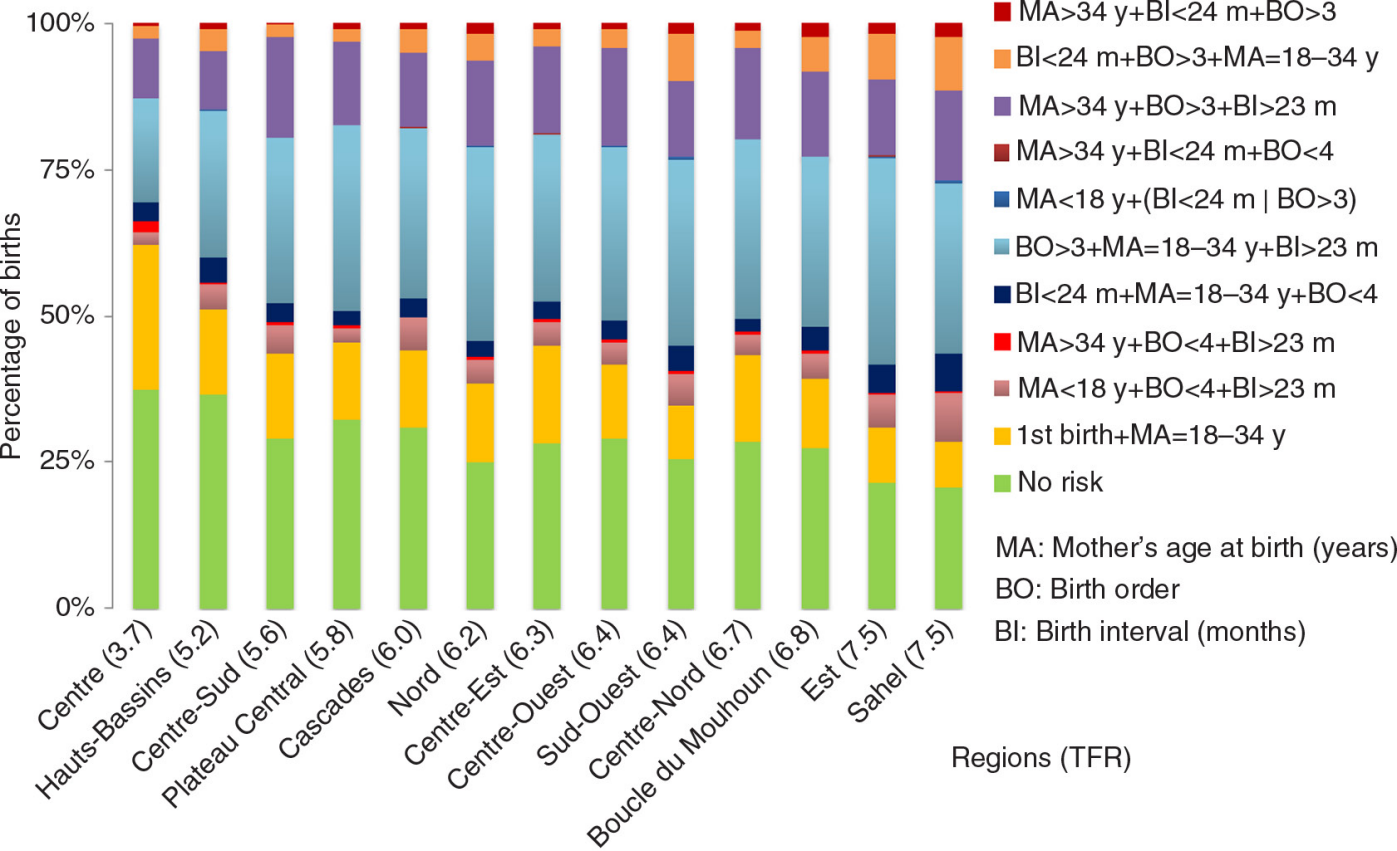
Distribution of births by risk factors and by region (total fertility rate: TFR) in 2010 in Burkina Faso.

Figure 2 presents the distribution of births classified as high risk, by place of residence and the change over time. For each year, we noted that the proportion of high-risk births was larger in rural areas than urban areas. Nearly 48.7% of births in 1998 in urban areas were in at least one high-risk category, while this proportion dropped to 40.7% in 2010. Conversely, 66% of rural births in 1998 were high risk compared with 61.7% in 2010. The national trends are more reflective of the rural trends, given that 84.5 and 77.3% of the population was rural in 1996 and 2006, respectively (24). The regional distribution of birth risk factors was examined (see Fig. 3). As expected, regions with a low TFR had a higher percentage of births with no risk compared with regions having a higher TFR. The percentage of high-risk births was lowest in the Centre and Hauts-Bassins regions and highest in the Sahel and Est regions. Consistent with expectations, the regions with the highest mCPRs had the lowest percentages of high-risk births.

#### Relationships between modern contraceptive use, high-risk births, and under-five child mortality

In general, there was a strong association between factors for high-risk birth and under-five mortality. Births belonging to any high-risk category had an under-five mortality rate of 142.0 deaths per 1,000 live births compared to 106.0 for births in the ‘unavoidable risk’ category and 101.7 for births classified as ‘not in any high-risk category’ (see Table 4). Among the high-risk categories, the mortality rate was 155.1 for multiple high-risk births compared with 134.9 for single high-risk births. The births that combined the risk factors of late birth, short birth spacing, and high birth parity had an under-five mortality rate of 270.1 per 1,000 live births compared with 212.1 for births that had short birth interval and high birth parity. Short birth spacing had the highest under-five mortality rate (190.0 per 1,000 live births) of any single high-risk factor. This factor also contributed to mortality in four out of the five multiple high-risk categories.

**Fig. 4:**
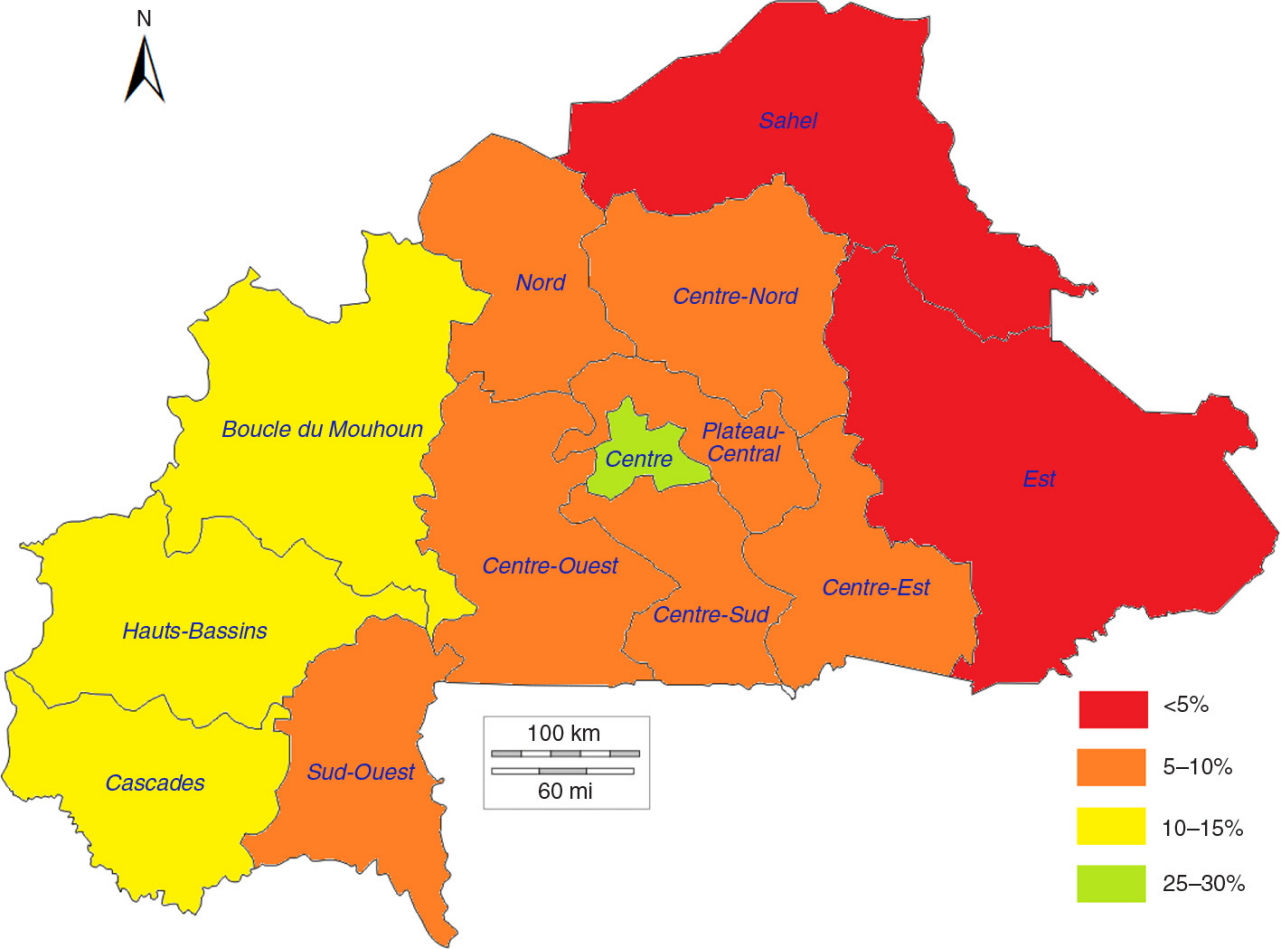
Modern contraceptive prevalence rates by region in 2003 in Burkina Faso.

**Fig. 5:**
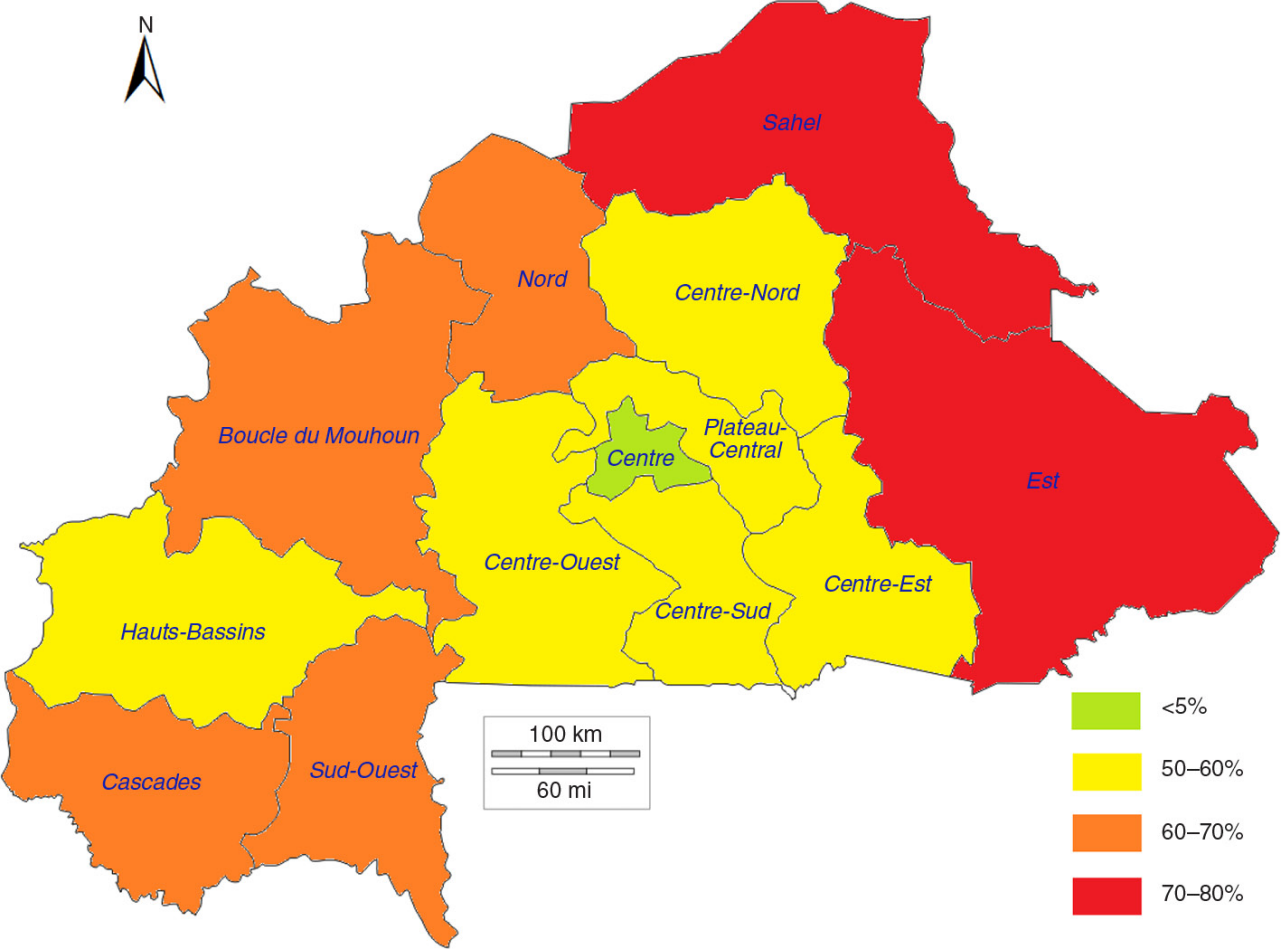
High-risk births distribution by region among children born between 2003 and 2008 in Burkina Faso.

**Fig. 6:**
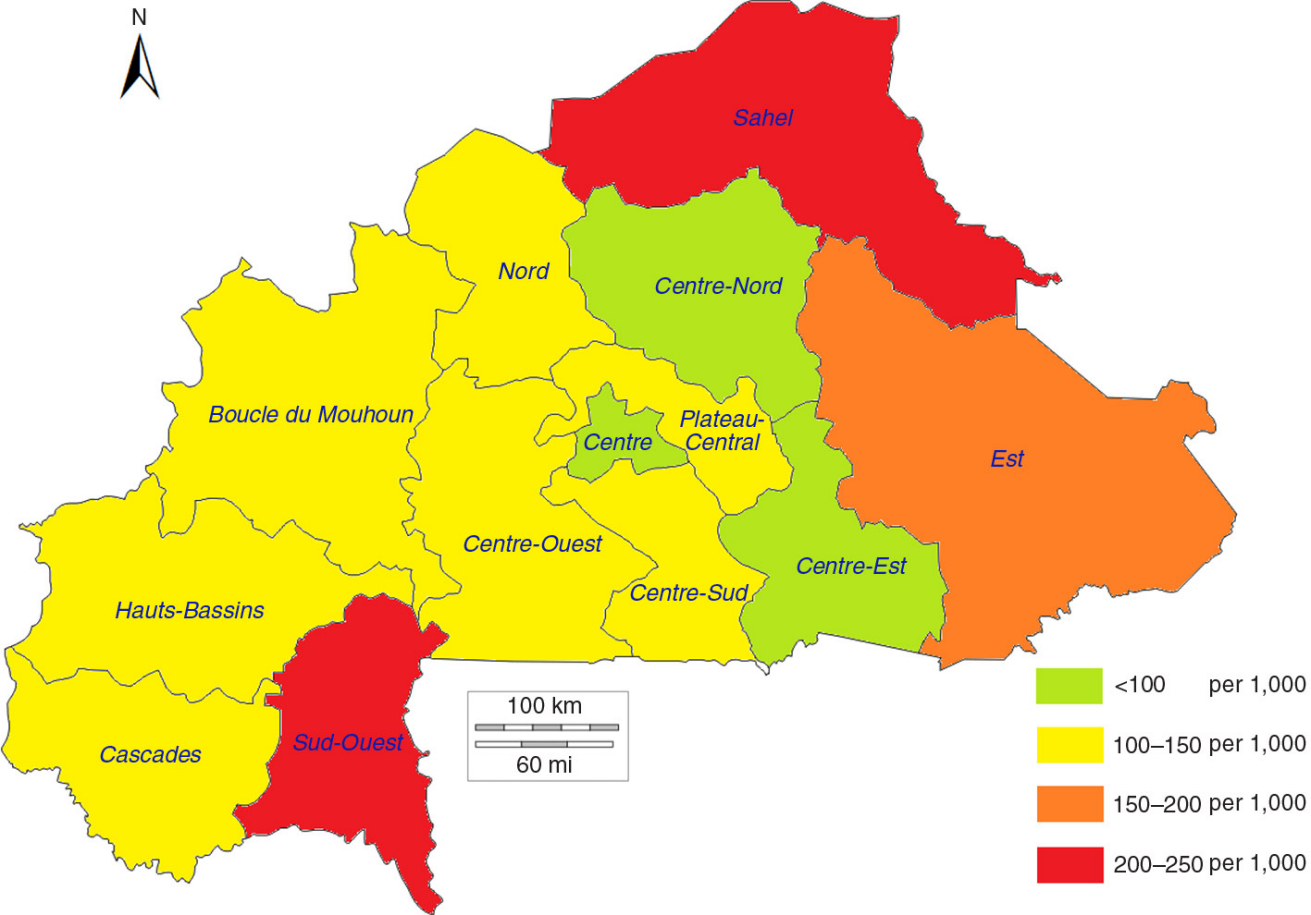
Under-five mortality rates by region among children born between 2003 and 2008 in Burkina Faso.

Figures 4–6 show a strong negative relationship of modern contraceptive use with the distribution of high-risk births and under-five mortality, where the regions characterised by low contraceptive use had the highest level of high-risk births and deaths of children, and vice versa. The Sahel region, with an mCPR of 3.5% in 2003, had 72.8% of births between 2003 and 2008 identified as high-risk and an under-five mortality rate of 205.0 per 1,000 live births. The mCPR, high-risk birth rates, and child mortality rates showed similar patterns in the Est (2.5%, 70.4%, and 157.8 per 1,000, respectively) and Sud-Ouest (7.5%, 65.0%, and 217.6 per 1,000, respectively) regions. The situation was reversed in the more urban Centre and Hauts-Bassins regions, where the comparatively higher mCPRs were associated with lower percentages of high-risk births and lower under-five mortality rates.

### Multivariate analyses

#### Extent to which high-risk birth factors are associated with modern contraceptive use

After adjusting for all covariates, high birth parity was not a significant predictor of contraceptive use in rural areas, but it was a predictor in urban areas and at the national level (see Table 5). In urban areas, the odds of women with parity higher than three children using contraception was more than twice that of women with just one child (aOR=2.17, 95% CI: 1.10–4.27), while national aOR was 1.49 (95% CI: 1.02–2.17). Rural women who experienced a short birth spacing for the latest childbirth were more likely to use modern contraception (aOR=1.61, 95% CI: 1.20–2.17) afterwards than those who had births spaced by more than 35 months. In urban areas, the latter group of women reported significantly greater contraceptive use compared to women who had birth intervals of between 24 and 35 months. We did not find any significant association between modern contraceptive use and the mother's age at childbirth, after adjusting the odds ratios.

**Table 5:** Adjusted odds ratios from logistic regression models of modern contraceptive use by women in a union with a child born in the preceding 5 years in Burkina Faso

	Model 1 – rural area			Model 2 – urban area			Model 3 – nationwide		
			
Risk birth factors and other background characteristics	aOR	95% CI		aOR	95% CI		aOR	95% CI	
Mother's age at birth									
<18	0.82	(0.41–1.63)	ns	1.08	(0.33–3.57)	ns	0.91	(0.51–1.62)	ns
18–34	1.12	(0.78–1.61)	ns	1.42	(0.69–2.92)	ns	1.19	(0.86–1.66)	ns
35–49	1.00	(ref.)		1.00	(ref.)		1.00	(ref.)	
Parity/birth order									
1	1.00	(ref.)		1.00	(ref.)		1.00	(ref.)	
2–3	1.22	(0.85–1.76)	ns	1.88	(1.25–2.84)	***	1.42	(1.08–1.87)	**
>3	1.21	(0.78–1.89)	ns	2.17	(1.10–4.27)	**	1.49	(1.02–2.17)	**
Birth interval									
First birth	n/a			na			na		
9–23 months	1.61	(1.20–2.17)	***	0.94	(0.50–1.77)	ns	1.41	(1.07–1.85)	**
24–35 months	0.98	(0.79–1.21)	ns	0.70	(0.52–0.95)	**	0.89	(0.75–1.06)	ns
36+ months	1.00	(ref.)		1.00	(ref.)		1.00	(ref.)	
Missing	2.63		ns	0.67	(0.10–4.56)	ns	0.75	(0.09–6.06)	ns
Sons born within preceding 5 years									
0	1.00	(ref.)		1.00	(ref.)		1.00	(ref.)	
1	1.34	(1.07–1.67)	**	1.09	(0.77–1.56)	ns	1.24	(1.02–1.49)	**
2–3	1.16	(0.79–1.73)	ns	0.88	(0.47–1.64)	ns	1.06	(0.76–1.49)	ns
Daughters born within preceding 5 years									
0	1.00	(ref.)		1.00	(ref.)		1.00	(ref.)	
1	1.16	(0.91–1.47)	ns	1.11	(0.79–1.56)	ns	1.13	(0.93–1.38)	ns
2–3	1.20	(0.80–1.80)	ns	0.95	(0.49–1.86)	ns	1.09	(0.77–1.55)	ns
Time since last birth									
<1 year	0.20	(0.15–0.28)	***	0.26	(0.16–0.40)	***	0.22	(0.17–0.29)	***
1–2 years	0.83	(0.67–1.03)	*	0.75	(0.55–1.01)	*	0.81	(0.68–0.97)	**
3+ years	1.00	(ref.)		1.00	(ref.)		1.00	(ref.)	
Told about FP at health facility?									
No	1.00	(ref.)		1.00	(ref.)		1.00	(ref.)	
Yes	3.17	(2.52–3.99)	***	2.21	(1.63–3.00)	***	2.81	(2.34–3.38)	***
Did not visit health facility	0.76	(0.58–0.99)	**	0.77	(0.53–1.12)	ns	0.75	(0.60–0.93)	***
Postnatal check at health facility?									
No	1.00	(ref.)		1.00	(ref.)		1.00	(ref.)	
Yes	1.04	(0.80–1.36)	ns	1.19	(0.81–1.74)	ns	1.10	(0.89–1.37)	ns
Missing	1.01	(0.60–1.71)	ns	0.84	(0.43–1.63)	ns	0.98	(0.66–1.47)	ns
Location of residence									
Urban	–			–			1.26	(0.99–1.60)	*
Rural	–			–			1.00	(ref.)	
Administrative region									
Boucle du Mouhoun	0.38	(0.23–0.62)	***	1.34	(0.65–2.74)	ns	0.69	(0.46–1.03)	*
Cascades	0.36	(0.18–0.68)	***	0.71	(0.41–1.21)	ns	0.58	(0.36–0.93)	**
Centre	1.00	(ref.)		1.00	(ref.)		1.00	(ref.)	
Centre-Est	0.38	(0.21–0.67)	***	0.69	(0.44–1.09)	ns	0.60	(0.40–0.88)	***
Centre-Nord	0.44	(0.28–0.71)	***	0.82	(0.43–1.57)	ns	0.69	(0.46–1.03)	*
Centre-Ouest	0.36	(0.20–0.66)	***	0.78	(0.48–1.27)	ns	0.60	(0.38–0.94)	**
Centre-Sud	0.73	(0.48–1.12)	ns	0.92	(0.51–1.65)	ns	1.09	(0.77–1.55)	ns
Est	0.50	(0.31–0.82)	***	1.89	(0.75–4.79)	ns	0.85	(0.56–1.29)	ns
Hauts-Bassins	0.86	(0.54–1.39)	ns	1.17	(0.73–1.85)	ns	1.24	(0.86–1.80)	ns
Nord	0.40	(0.26–0.62)	***	0.96	(0.57–1.64)	ns	0.66	(0.46–0.95)	**
Plateau Central	0.45	(0.28–0.72)	***	0.54	(0.27–1.10)	*	0.67	(0.44–1.01)	*
Sahel	0.65	(0.33–1.28)	ns	1.25	(0.58–2.71)	ns	0.94	(0.53–1.67)	ns
Sud-Ouest	0.34	(0.12–1.01)	*	0.84	(0.45–1.57)	ns	0.64	(0.35–1.17)	ns
Wealth quintile									
Poorest	1.00	(ref.)		1.00	(ref.)		1.00	(ref.)	
Poorer	1.31	(1.01–1.69)	**	1.83	(0.95–3.51)	*	1.30	(1.02–1.66)	**
Middle	1.25	(0.96–1.61)	*	2.16	(0.97–4.84)	*	1.30	(1.02–1.66)	**
Richer	1.95	(1.48–2.56)	***	2.96	(1.59–5.51)	***	2.08	(1.62–2.68)	***
Richest	3.93	(2.71–5.70)	***	4.88	(2.55–9.32)	***	3.55	(2.63–4.80)	***
Educational attainment									
No education	1.00	(ref.)		1.00	(ref.)		1.00	(ref.)	
Primary	1.77	(1.38–2.28)	***	1.12	(0.82–1.53)	ns	1.48	(1.21–1.80)	***
Secondary or greater	5.53	(3.43–8.93)	***	2.24	(1.57–3.21)	***	3.00	(2.24–4.02)	***
Ethnicity									
Mossi	1.00	(ref.)		1.00	(ref.)		1.00	(ref.)	
Bobo	2.15	(1.43–3.23)	***	1.29	(0.82–2.03)	ns	1.88	(1.37–2.58)	***
Peulh	0.54	(0.33–0.87)	**	0.94	(0.52–1.69)	ns	0.62	(0.42–0.90)	**
Gourmantche	0.74	(0.47–1.16)	ns	0.58	(0.17–1.98)	ns	0.74	(0.49–1.10)	ns
Gourounsi	0.72	(0.38–1.37)	ns	1.51	(0.74–3.06)	ns	0.85	(0.53–1.34)	ns
Lobi/Dagara	1.53	(0.55–4.28)	ns	1.26	(0.69–2.29)	ns	1.28	(0.74–2.23)	ns
Bissa	1.30	(0.80–2.09)	ns	1.13	(0.65–1.95)	ns	1.24	(0.88–1.76)	ns
Other ethnicity	1.08	(0.68–1.72)	ns	0.92	(0.52–1.62)	ns	0.96	(0.67–1.36)	ns
Other country	0.71	(0.16–3.03)	ns	1.01	(0.40–2.55)	ns	0.87	(0.37–2.05)	ns
Religion									
Muslim	1.00	(ref.)		1.00	(ref.)		1.00	(ref.)	
Catholic	1.16	(0.92–1.46)	ns	1.44	(1.02–2.03)	**	1.28	(1.06–1.55)	**
Protestant	2.22	(1.54–3.21)	***	0.97	(0.59–1.58)	ns	1.78	(1.30–2.45)	***
Traditional	0.55	(0.37–0.82)	***	0.54	(0.24–1.19)	ns	0.56	(0.39–0.81)	***
Mother's age at time of survey	1.03	(0.91–1.18)	ns	0.95	(0.76–1.18)	ns	0.99	(0.89–1.11)	ns
Mother's age squared at time of survey	1.00	(1.00–1.00)	ns	1.00	(1.00–1.00)	ns	1.00	(1.00–1.00)	ns

* *p*<0.10,

** *p*<0.05,

*** *p*<0.01. ns, not significant; na, not applicable; aOR, adjusted odds ratio; CI, confidence interval; ref., value of reference.

#### Modern contraception use in relation to gender preference, time since childbirth, and contact with the health system

Overall, the analyses demonstrated that the number of daughters born and still alive in the 5 years preceding the time of survey was not associated with the probability of using modern contraception (see Table 5). However, in rural areas and nationally, women who had one son during the last 5 years were more likely (aOR=1.34, 95% CI: 1.07–1.67 in rural areas; aOR=1.24, 95% CI: 1.02–1.49 nationally) to use a contraceptive method than those without a male child.

The odds of using modern contraception were significantly lower for women who had given birth within the previous year (aOR=0.22, 95% CI: 0.17–0.29 nationally; aOR=0.20, 95% CI: 0.15–0.28 in rural areas; aOR=0.26, 95% CI: 0.16–0.40 in urban areas), as well as for women whose most recent childbirth was 1 to 2 years ago (aOR=0.81, 95% CI: 0.68–0.97 nationally).

Post-natal visits within 2 months of childbirth was not significant in predicting use of contraception. In contrast, whether the woman visited a health facility within the last 12 months and was told about FP was significantly associated (*p*<0.01), nationally as well as in urban and rural areas, with odds of using contraception, two to three times higher than that of women who were not told about FP at a health facility. Moreover, modern contraceptive use was lower for women who did not have a health facility visit within the last 12 months nationally and in rural areas.

#### Place of residence and cultural and socio-economic characteristics as determinants of modern contraception use

Modern contraceptive use was more likely in urban areas (aOR=1.26, 95% CI: 0.99–1.60) compared to rural areas in Burkina Faso (see Table 5). Considering the total population in each region, modern contraceptive use was lower in Centre-Est (aOR=0.60, 95% CI: 0.40–0.88), Centre-Ouest (aOR=0.60, 95% CI: 0.38–0.94), and Nord (aOR=0.66, 95% CI: 0.46–0.95), compared with the Centre region. After controlling for the urban/rural designation, modern contraceptive use was lower in the rural parts of most regions (excepting Hauts-Bassins, Sahel, and Centre-Sud) with significantly lower use in Centre-Ouest (aOR=0.36, 95% CI: 0.20–0.66), Centre-Est (aOR=0.38, 95% CI: 0.21–0.67), and Nord (aOR=0.40, 95% CI: 0.26–0.62). For the model using urban data, modern contraceptive use was not significantly different between regions.

Modern contraceptive use generally increased with increasing household wealth and women's education attainment in all three models – urban, rural, and national. For instance, the odds of using modern contraception were more than three times higher for women in the richest household wealth quintile compared to women in the poorest wealth quintile in all models. Similarly, contraceptive use was significantly higher in women with at least a secondary education compared to women with no education, while nationally the odds of a more educated woman using modern contraception were about three times higher than those of an uneducated woman. While both were statistically significant (*p<*0.01), it was noted that the odds ratio for women with at least a secondary education in rural areas (aOR=5.53, 95% CI: 3.43–8.93) was higher than that of women in urban areas (aOR=2.24, 95% CI: 1.57–3.21), suggesting that the relationship between education and contraception use may be stronger in rural than urban areas. It should be noted that only 1.8% of the rural women were identified as having secondary or higher education, compared with 22.8% in urban areas and 5.5% overall.

Women from the Bobo ethnic group were more likely to use modern contraception (aOR=1.88, 95% CI: 1.37–2.58 nationally) than women from the Mossi ethnic group (the reference ethnic group making up 51.6% of the total women in the dataset nationally). In contrast, women in the Peulh ethnic group were much less likely to use modern contraception (aOR=0.62, 95% CI: 0.42–0.90 nationally). Religious affiliation was also a significant predictor of modern contraceptive use nationally. Compared to the Muslim religion group (63.9% of the total women in the data set), Catholic (aOR=1.28, 95% CI: 1.06–1.55) and Protestant (aOR=1.78, 95% CI: 1.30–2.45) women were more likely to be users. Women associated with traditional religions were less likely to be using modern contraception (aOR=0.56, 95% CI: 0.39–0.81).

## Discussion

Even though modern contraceptive use represents one of the most studied issues in reproductive health, few things are known about its associations with birth risk and child mortality at both the national and sub-national levels. In this respect, our results clearly showed a strong relationship between modern contraceptive use and the distribution of high-risk births, as low contraceptive use was associated with the highest rates of birth risk and child mortality. Although these findings applied to most regions in Burkina Faso, the issue was more noticeable in the Sahel, Est, and Sud-Ouest regions. We also found a strong relationship between child mortality and factors for high-risk birth, such as short birth intervals (<24 months). This may indicate that programmes for improving the percentage of demand satisfied for modern methods of contraception in this country may result not only in a decline in unplanned pregnancies, but may also change the percentage and distribution of high-risk births. Improving modern contraceptive use can result in greater birth spacing and may lead to reductions in child mortality. This opportunity exists throughout Burkina Faso, as the percentage of demand for modern contraceptive methods satisfied was only 37.2% nationally, ranking between 25.2 and 53.2% in various regions in the 2010 DHS survey.

While women generally had a high level of knowledge about modern methods of contraception (98%) (2), the overall usage in 2010 was comparatively low at 15.4%, maybe a reflection of combined lack of demand and barriers in supply and demand. Similar to previous studies (32, 6, 12), we confirmed nevertheless that modern contraceptive use was more likely in urban areas compared to rural areas in Burkina Faso. In addition, there was no significant increase in mCPR, especially where the prevalence was already higher in 2003. Data from the next DHS in Burkina Faso may facilitate understanding of whether this was just a short-term stall in mCPR increase.

The observation that modern contraceptive use was higher among wealthier households in both urban and rural areas after controlling for factors such as education may reflect issues of supply, demand, or affordability. Given DHS limitations for examining institutional and economic barriers to usage, further investigations are needed to determine what is driving these issues. A key insight from this research involves the critical relationship between women's interactions with the health system and the use of modern contraception. The importance of the health system was emphasised by the observation that the odds of using modern contraceptives were more than doubled for women who had been informed about FP during a health facility visit in the last 12 months, as compared to women who had visited a facility but were not informed. This difference may also reflect the current model of delivery of FP services, which are mainly provided through health facilities as opposed to being community-based. The results suggest, however, that programming that integrates FP information into antenatal and postnatal care health facility visits is likely to have a substantial impact on contraceptive prevalence rates. In the light of that, it should be worthwhile to take account of and leverage this integration opportunity in future programmes.

The results suggested that ethnic group and religious affiliation were significant cultural factors in predicting the use of modern contraception. After controlling for other covariates, lower contraceptive use was observed for women belonging to the Peulh ethnic group and among those who were Muslim or in traditional/animist religions. Qualitative analysis of data on perceptions, values, and cultural beliefs may need to be performed to better understand the barriers and the potential unmet needs for FP among those populations. It is important that FP messaging be sensitive to the diverse cultural and ethnic groups within Burkina Faso.

As previously mentioned, high-risk births may represent a potential increased mortality risk to both mothers and children, which we hoped to address specifically. However, available data did not permit analysis of the relationship between mCPR or birth risk and maternal mortality, since there were no detailed data on women who died. The key limitations of this study related to the data source itself, the DHS and, more generally, retrospective surveys. Much of the data for this survey was based on recall, including the enumeration of births, reporting of early neonatal deaths, recollection of timing of facility visits, and corresponding exposure to FP information. Recall bias and error may have resulted in underreporting of early neonatal death cases or childbirths, or in inaccuracy of timing related to reported events. In addition to recall issues, this analysis focused on births that occurred within the 5 years preceding the survey. As a result, it is possible that changes occurred to the mother's individual, household, or ecological characteristics during the time gap between the survey and the birth being used for the analysis. Specifically, one could envision situations such as the birth being referenced occurring during a time when the mother was living in a rural area but, at the time of the survey, she was recorded as living in an urban area. Future research could explore the influence of this issue through sensitivity analysis around the time window used for previous births.

## Conclusion

In Burkina Faso, modern contraception prevalence remains low, despite a high level of knowledge about FP. Moreover, the regional heterogeneity highlighted can reflect an issue of equity in terms of FP coverage. Increasing the demand satisfied by modern contraception would reduce the percentage of births that are high risk and probably result in reducing child mortality through a variety of mechanisms, including achieving a lower percentage of short-interval births. Contact with the health system is a critical opportunity for promoting modern contraceptive use and, more broadly, for FP programming. FP programming that is integrated into routine care as well as antenatal and postnatal visits is likely to have significant impact, enabling Burkina Faso to reduce the unmet need for FP and enabling women to fully exercise their reproductive rights.

## Supplementary Material

Trends and patterns of modern contraceptive use and relationships with high-risk births and child mortality in Burkina Faso
