# Supplementary material for: Trends and patterns of modern contraceptive use and relationships with high-risk births and child mortality in Burkina Faso
Source: Glob Health Action. 2015 Nov 9;8:10.3402/gha.v8.29736. doi: 10.3402/gha.v8.29736 (PMC4642359; doi:10.3402/gha.v8.29736)
Supplement: Trends and patterns of modern contraceptive use and relationships with high-risk births and child mortality in Burkina Faso [file GHA-8-29736-s001.pdf]

Supplementary File to Maïga et al: Trends and patterns of modern contraceptive use and relationships with high-risk births and child mortality in Burkina Faso.
***Global Health Action* 2015; 8:29736.**

Table Appendix 1: Proportions (%) and numbers (N) of background characteristics used in logistic regressions models

Background characteristics	Rural area			Urban area			Country overall		
	%	95% CI	N	%	95% CI	N	%	95% CI	N
**Mother's age at birth**									
<18	3.9	(3.5-4.5)	322	2.5	(1.9-3.3)	45	3.7	(3.3-4.1)	367
18-34	75.9	(74.8-77.0)	6203	83.3	(81.3-85.2)	1496	77.3	(76.2-78.3)	7698
35-49	20.1	(19.1-21.2)	1644	14.2	(12.4-16.1)	254	19.1	(18.1-20.0)	1898
**Parity / Birth order**									
1	14.2	(13.4-15.1)	1163	22.9	(20.5-25.4)	411	15.8	(14.9-16.7)	1573
2-3	30.5	(29.3-31.6)	2488	43.1	(40.3-45.9)	773	32.7	(31.6-33.9)	3261
>3	55.3	(54.0-56.6)	4518	34.1	(31.3-36.9)	611	51.5	(50.1-52.9)	5130
**Birth interval**									
First birth	14.2	(13.4-15.1)	1163	22.9	(20.5-25.4)	411	15.8	(14.9-16.7)	1573
9-23 months	9.3	(8.5-10.2)	759	5.7	(4.6-7.0)	103	8.6	(7.9-9.4)	861
24-35 months	32.5	(31.3-33.8)	2658	20.2	(18.2-22.3)	362	30.3	(29.1-31.5)	3020
36+ months	43.9	(42.4-45.3)	3585	50.8	(48.1-53.4)	911	45.1	(43.8-46.4)	4496
Missing	0.1	(0.0-0.2)	5	0.5	(0.2-1.2)	8	0.1	(0.1-0.3)	13
**Sons born within 5 preceding years**									
0	41.1	(39.9-42.3)	3360	44.2	(41.9-46.6)	794	41.7	(40.6-42.8)	4154
1	48.9	(47.6-50.1)	3991	49.5	(47.2-51.7)	888	49.0	(47.9-50.0)	4879
2-3	10.0	(9.3-10.8)	818	6.3	(5.2-7.6)	113	9.3	(8.7-10.0)	931
**Daughters born within 5 preceding years**									
0	42.2	(41.0-43.5)	3451	45.6	(43.3-48.0)	819	42.9	(41.8-44.0)	4270
1	47.8	(46.7-49.0)	3908	47.8	(45.6-50.1)	859	47.8	(46.8-48.9)	4767
2-3	9.9	(9.2-10.7)	810	6.5	(5.3-8.0)	117	9.3	(8.7-10.0)	927
**Time since last birth**									
<1y	30.5	(29.4-31.6)	2493	26.1	(23.7-28.7)	468	29.7	(28.7-30.7)	2962
1-2y	50.5	(49.2-51.7)	4122	48.2	(45.6-50.9)	866	50.1	(48.9-51.2)	4987
3y+	19.0	(18.1-20.0)	1554	25.7	(23.0-28.5)	461	20.2	(19.3-21.2)	2015
**Told about FP at health facility (H.F.)**									
No told	37.7	(35.5-40.0)	3081	41.0	(37.5-44.6)	736	38.3	(36.4-40.3)	3817
Yes told	36.2	(33.8-38.7)	2958	37.5	(34.2-40.9)	673	36.4	(34.4-38.5)	3631
Not visited H.F.	26.1	(24.2-28.0)	2129	21.5	(19.2-24.0)	386	25.2	(23.6-26.9)	2515
**Postnatal check in health facility**									
No	18.2	(16.1-20.5)	1485	13.8	(11.3-16.7)	247	17.4	(15.6-19.3)	1733
Yes	78.3	(76.0-80.5)	6398	80.5	(76.7-83.7)	1444	78.7	(76.7-80.6)	7843
Missing	3.5	(2.8-4.3)	285	5.8	(4.4-7.5)	103	3.9	(3.3-4.6)	389
**Place of residence**									
Urban	0.0	-	0	100.0	-	1795	18.0	(15.0-21.5)	1795
Rural	100.0	-	8169	0.0	-	0	82.0	(78.5-85.0)	8169

Table Appendix 1 (continued)

Background characteristics	Rural area			Urban area			Country overall		
	%	95% CI	N	%	95% CI	N	%	95% CI	N
**Administrative region**									
Boucle de Mouhoun	13.2	(9.6-17.8)	1077	4.0	(2.0-7.6)	71	11.5	(8.5-15.4)	1148
Cascades	3.6	(2.4-5.3)	291	4.9	(2.4-9.7)	88	3.8	(2.7-5.4)	379
Centre	2.1	(1.2-3.7)	174	42.1	(32.7-52.1)	756	9.3	(7.0-12.4)	930
Centre-Est	8.2	(5.7-11.9)	674	6.9	(3.9-11.8)	123	8.0	(5.8-11.0)	797
Centre-Nord	9.2	(6.6-12.8)	754	2.8	(1.4-5.5)	50	8.1	(5.9-11.0)	804
Centre-Ouest	8.6	(6.0-12.1)	700	5.0	(2.7-9.2)	90	7.9	(5.7-10.9)	790
Centre-Sud	5.3	(3.6-7.7)	435	2.4	(1.2-4.6)	42	4.8	(3.4-6.8)	477
Est	12.4	(9.0-16.9)	1014	3.0	(1.5-5.9)	53	10.7	(7.9-14.4)	1068
Hauts-Bassins	9.2	(6.2-13.3)	748	18.9	(12.3-27.9)	340	10.9	(8.1-14.5)	1088
Nord	8.4	(5.9-11.8)	684	4.1	(2.2-7.5)	73	7.6	(5.5-10.4)	757
Plateau Central	5.3	(3.7-7.6)	434	1.8	(0.8-4.0)	32	4.7	(3.3-6.6)	466
Sahel	10.1	(7.2-13.8)	823	2.1	(1.0-4.3)	37	8.6	(6.3-11.8)	860
Sud-Ouest	4.4	(3.0-6.5)	362	2.1	(1.1-4.0)	38	4.0	(2.8-5.7)	400
**Wealth quintile**									
Poorest	23.4	(21.3-25.6)	1910	1.9	(1.2-3.0)	34	19.5	(17.7-21.5)	1944
Poorer	25.4	(23.9-27.0)	2075	3.0	(2.0-4.5)	53	21.4	(19.9-22.9)	2129
Middle	25.1	(23.5-26.8)	2049	5.6	(4.0-7.7)	100	21.6	(20.1-23.1)	2149
Richer	21.3	(19.5-23.3)	1740	20.4	(16.7-24.7)	366	21.1	(19.5-22.9)	2106
Richest	4.8	(3.8-6.1)	394	69.2	(63.3-74.5)	1242	16.4	(14.0-19.1)	1636
**Education attainment**									
No education	90.2	(89.2-91.2)	7371	52.7	(49.1-56.3)	946	83.5	(81.8-85.0)	8317
Primary	8.0	(7.2-8.9)	654	24.6	(22.2-27.1)	441	11.0	(10.0-12.0)	1095
Secondary+	1.8	(1.4-2.1)	144	22.8	(19.6-26.2)	408	5.5	(4.7-6.6)	552
**Ethnicity**									
Mossi	49.2	(44.7-53.7)	4018	62.3	(56.5-67.8)	1119	51.6	(47.7-55.4)	5137
Bobo	9.6	(7.2-12.8)	786	10.8	(7.6-15.0)	193	9.8	(7.7-12.5)	980
Peulh	13.0	(10.5-15.9)	1061	4.8	(3.5-6.5)	86	11.5	(9.5-13.9)	1147
Gourmantché	9.9	(7.1-13.6)	810	2.2	(1.1-4.4)	40	8.5	(6.2-11.6)	850
Gourounsi	4.4	(2.9-6.7)	362	3.5	(2.3-5.3)	63	4.3	(3.0-6.1)	425
Lobi/Dagara	4.3	(3.0-6.2)	354	3.6	(2.5-5.1)	64	4.2	(3.1-5.7)	418
Bissa	3.8	(2.4-6.0)	311	3.7	(2.2-6.0)	66	3.8	(2.6-5.5)	376
Other ethnicity	5.5	(3.9-7.8)	453	7.5	(5.3-10.5)	135	5.9	(4.5-7.7)	587
Other country	0.2	(0.1-0.5)	14	1.6	(1.1-2.6)	30	0.4	(0.3-0.7)	44
**Religion**									
Muslim	63.6	(59.9-67.2)	5194	65.1	(60.8-69.2)	1169	63.9	(60.7-66.9)	6363
Catholic	19.4	(17.2-21.8)	1584	27.0	(23.5-30.8)	485	20.8	(18.8-22.8)	2069
Protestant	6.2	(4.9-7.9)	510	5.8	(4.2-8.0)	105	6.2	(5.0-7.5)	614
Traditional	10.8	(8.8-13.2)	882	2.0	(1.1-3.8)	36	9.2	(7.5-11.2)	918
**Mother's age at time of survey**	29.5	(29.3-29.7)	(mean)	29.2	(28.8-29.5)	(mean)	29.4	(29.2-29.6)	(mean)
**Mother's age squared at time of survey**	920.8	(908.4-933.2)	(mean)	893.4	(870.9-915.8)	(mean)	915.9	(904.9-926.8)	(mean)
**Total not weighted**	100.0		8169	100.0		1795	100.0		9964
**Total weighted**	100.0		8307	100.0		1825	100.0		10132

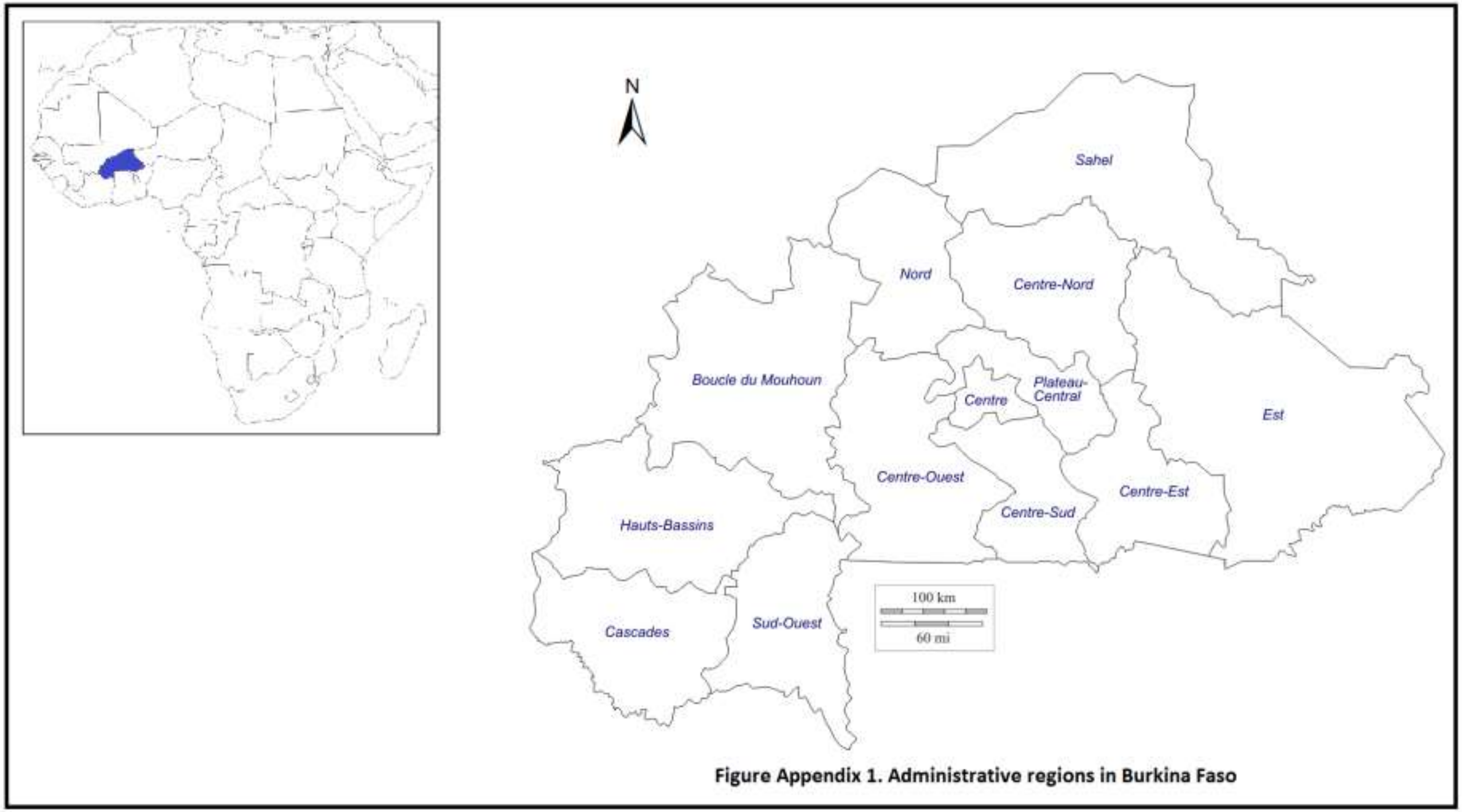

Figure Appendix 1. Administrative regions in Burkina Faso
